# Is outcome of anterior cervical discectomy for cervical radiculopathy influenced by securing the intervertebral cage?

**DOI:** 10.1016/j.bas.2026.106039

**Published:** 2026-04-12

**Authors:** Azra Gül, Yi Lu, Hasan A. Zaidi, Jessica Grace, Daniela Limbania, Timothy R. Smith, Caroline MW. Goedmakers, Carmen Vleggeert-Lankamp

**Affiliations:** a Department of Neurosurgery, Leiden University Medical Centre, Leiden, the Netherlands; b Computational Neuroscience Outcomes Center, Department of Neurosurgery, Brigham and Women's Hospital, Harvard Medical School, Boston, MA, USA; c Department of Endocrinology Beth Israel Deaconess Medical Center, Boston, MA, USA; d Department of Neurosurgery, Boston Medical Center, Boston, MA, USA; e Department of Neurosurgery, Spaarne Gasthuis, Haarlem/Hoofddorp, the Netherlands

**Keywords:** ACDF, Cervical radiculopathy, VAS, Standalone cage

## Abstract

**Introduction:**

Anterior Cervical Discectomy and Fusion (ACDF) for cervical radiculopathy is commonly performed with a cage and plate in the United States, whereas in Europe, standalone cages are frequently used instead. Knowledge about difference in clinical outcome is scarce.

**Research question:**

Are there differences in clinical outcomes in patients undergoing ACDF using a cage with an anterior plate, a cage with integrated screws, or a standalone cage?

**Methods:**

570 patients with cervical radiculopathy were included: 414 received a cage with plate (US), 54 received cages with integrated screws (US) and 102 received a standalone cage (86% Netherlands). The clinical outcomes Visual Analogue Scale arm and neck pain, and pain interference were assessed at baseline, 3, 6, 12 and 24 months after inclusion, and analysed using linear mixed-effects models. Furthermore, the Minimal Clinical Important Difference was assessed and to explore individual patient-level outcome, cut-off values for clinical success were utilized.

**Results:**

Baseline clinical parameters were comparable in the three groups. Linear mixed-effects models revealed no statistically significant differences in clinical outcome over time. However, dichotomized clinical success rates for neck pain were significantly higher in the standalone group (62.9% at 6 months, 79.3% at 12 months, 85.7% at 24 months), compared to the cage with plate group (59.5%, 61.2%, 58.9%) and the integrated screws group, which showed more variability (58.3%, 26.7%, 44.4%).

**Discussion and conclusion:**

No substantial differences were observed at the group mean-level, however, the standalone cage group demonstrated higher individual-level success-rates, particularly regarding neck pain.

## Highlights

• ACDF cage fixation strategies vary between the United States and Northern Europe.
• Mixed-effects models demonstrate comparable mean improvements in clinical outcomes.
• Dichotomized success analyses show higher neck pain success with standalone cages

## 1 Introduction

### 1.1 Background

Anterior cervical discectomy and fusion (ACDF) is the most commonly performed surgical procedure for the treatment of cervical radiculopathy caused by disc herniation. It is widely regarded as the gold standard due to its reliable patient-reported outcomes and favorable risk profile (Rao et al., 2006; Ban et al., 2016; Chang et al., 2022). While ACDF is performed worldwide, it remains controversial whether the intervertebral cage should be secured using a plate or using integrated screws. Variation persists, particularly between surgeons from the United States and Europe.

In the United States ACDF is commonly performed with a cage and plate (Pereira et al., 2013; De la Garza-Ramos et al., 2016; Safdar et al., 2023), whereas in Northern Europe, standalone cages are frequently used instead (Vleggeert-Lankamp et al., 2019; Kotkansalo et al., 2019; Simões de Souza et al., 2024; Buwaider et al., 2025). A third, less frequently employed alternative is the cage with integrated screws (Takhelmayum et al., 2019; Vaishnav et al., 2019). Despite the widespread application of these three techniques, no studies have simultaneously compared the clinical outcomes of these three constructs in a single-level ACDF procedure. A previous systematic review by Elias et al., assessed clinical outcome parameters of standalone cages versus cages with plates. They were not able to detect a significant difference between the groups, presumably due to the limited number of included patients in all of the evaluated 41 studies, the large variance in follow up times, and the distinct surgical indications with accompanying spread in outcome parameters. The authors conclude with an emphasis on the necessity for larger studies with longer follow-up period to draw definitive conclusions (Elias et al., 2024).

An ACDF procedure involves the removal of the degenerated or herniated intervertebral disc to eventually decompress the nerve root, and is followed by the placement of a cage to maintain foraminal height and avoid kyphosis of the target level. The main purpose of placing a plate is to secure the position of the cage, thereby preventing its displacement and provide biomechanical segmental rigidity to increase the chances of fusion across the interbody cage. Cages with integrated screws were developed as an alternative to simplify the procedure. Both a cage with plate and a cage with integrated screws carry the potential risk of loosening and pull-out over time, which can lead to complications such as damage to the overlying esophagus. Additional potential disadvantages are higher costs and prolonged surgical time.

In contrast, a standalone cage is associated with lower rates of dysphagia, reduced operative time and minimized soft tissue disruption, which may further contribute to improved postoperative outcomes in selected patient populations (Nemoto et al., 2015; Sommaruga et al., 2021). Despite these advantages, many surgeons still prefer to use a plate out of concerns for potential cage migration, (which is however, a rare complication (Arif et al., 2024; Sharma and Sieg, 2022)), and due to its perceived benefits in promoting fusion and reducing the risk of cage subsidence and segmental kyphosis (Oliver et al., 2018). Therefore, the choice of surgical device is often shaped by a combination of clinical evidence, risk aversion and systemic reimbursement structures.

### 1.2 Objective of the study

The annual number of ACDF procedures in the United States is projected to increase from 153,288 in 2020 to 173,699 by 2040 (Neifert et al., 2020), underscoring the need for evidence-based decision-making in surgical technique selection. The present study aims to evaluate potential discrepancies in clinical outcomes over time among patients undergoing ACDF with a cage and plate, a cage with integrated screws, or a standalone cage. By systematically assessing differences in patient-reported outcomes between these three surgical groups, this study seeks to provide valuable insights to optimize treatment strategies and improve long-term patient outcomes.

## 2 Methods

### 2.1 Study design

A multi-center retrospective cohort study was conducted in two patients’ groups: patients who underwent an ACDF procedure between January 2019 and November 2024 in the Brigham and Woman's Hospital (BWH) in Boston (United States cohort) and patients who were included in the CASINO trial (the Netherlands cohort) between August 2012 and February 2021 (Gül et al.). All patients suffered from cervical radiculopathy due to a single level disc herniation. Approval was obtained from the institutional review board; informed consent from the BWH was waived due to the retrospective design of the study (IRB no. 2015P002352). Written informed consent was obtained from patients in the Netherlands cohort at the time of enrolment. Patients that were operated in Boston routinely complete patient-reported outcome measurement questionnaires as part of standard postoperative care and follow-up. Patients received standard PROMs questionnaires by mail at baseline, 3, 6, 12 and 24 months after surgery.

The CASINO trial (NTR3504) has been conducted as a prospective, multi-centre observational cohort study aiming to compare conservative to surgical treatment outcomes in patients with cervical radiculopathy caused by intervertebral disc herniation (Gül et al.).

### 2.2 Eligibility

The Boston study population was comprised of patients, aged 18 to 70 years old, with at least two months of radicular complaints prior to surgery, a minimum of six months of clinical and radiological follow-up, and an available post-operative x-ray examination. Patients with a history of prior spine surgery or those who underwent surgery for malignancy were excluded. Patients from the CASINO trial patients, aged 18 to 65 years old, with radicular signs and symptoms, in one or both arms (pain, paraesthesia or paresis in a specific nerve root distribution), for at least eight weeks and for whom conservative therapy (no physiotherapy or injections were prescribed) failed were eligible for inclusion.

### 2.3 Baseline characteristics

**Fig. 1:**
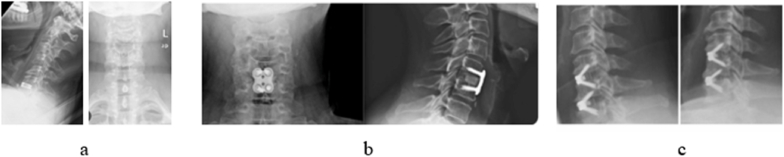
Standalone cage (a), cage with plate (b), cage with integrated screws (c).

The baseline demographic characteristics age, gender, smoking history and Body Mass Index (BMI) were collected for all patients. In the US cohort ‘anxiety/depression score’ and ‘alcohol consumption history’ were additionally available at baseline. The patient cohort was stratified based on whether patients received a standalone cage, cage with plate or cage with integrated screws during the ACDF procedure. Within the US cohort, the type of the implanted surgical device used during operation was scored on the postoperative radiographs, independently evaluated by three authors (AG, JG, DL) (Fig. 1). In the Netherlands cohort, every patient received a standalone cage.

### 2.4 Clinical outcomes

The clinical outcome parameters Visual Analogue Scale (VAS) arm pain and VAS neck pain were retrieved at baseline, 3, 6, 12 and 24 months after inclusion. The US cohort additionally contained data on pain interference on these timepoints.

#### 2.4.1 Smoking history

Smoking history was assessed by smoking behavior; patients could be classified as being a ‘non-smoker’ or ‘smoking less than 1 package a day’ or ‘more than 1 package a day’. Patients who had quit smoking more than six months prior to surgery were classified as part of the non-smoking group. Assessment of smoking behaviour was evaluated based on interviews prior to surgery (US cohort) and at the time of enrolment (Netherlands cohort). Moreover, in the US cohort the number of pack years was known.

#### 2.4.2 Anxiety/depression

Anxiety/Depression score within the US cohort is derived by averaging the scores from the PROMIS Anxiety and Depression item banks, with higher scores indicating worse outcomes. The anxiety scale evaluates fear, anxious misery, hyperarousal and psychosomatic symptoms such as a racing heart and dizziness and ranges from 40.3 to 81.6. The depression scale assesses negative mood, self-perception, social cognition and reduced positive affect and ranges from 41.0 to 79.4 (HealthMeasures, 2025a, 2025b). In both scales, a higher score represents a worse outcome.

#### 2.4.3 Alcohol consumption

Alcohol consumption history was evaluated in the US cohort through the Alcohol Use Disorders Identification Test- Consumption (AUDIT-C). The AUDIT-C consists of a three-item screening measurement ranging from 0 (non-drinkers) to 12 (possible alcohol dependence) (Simon et al., 2024).

#### 2.4.4 Pain interference

The PROMIS Pain Interference scale investigates the impact of pain on daily functioning, including social, cognitive, emotional, physical, and recreational activities and is known for its high responsiveness (Schuller et al., 2023). The PROMIS Pain Interference is part of the standard questionnaires after surgery to evaluate pain and ranges from 41.6 to 75.6 in which a lower score represents lower pain interference. In the Netherlands cohort, this was not available.

#### 2.4.5 VAS

The Visual Analogue Scale ranges from 0 (no pain) to 10 (most terrible pain I can imagine) and illustrates disabling pain in the arm or neck. Validity, reliability and responsiveness of the VAS have been previously demonstrated (Huskisson, 1974; Carlsson, 1983). Within the CASINO trial, the VAS scale ranged from 0 to 100 instead of 0-10, thus we converted this to merge the data.

### 2.5 MCID definition

Treatment effectiveness is frequently the primary objective in clinical trials; however, the full scope of patient benefits might not always be captured. Additionally analyzing the Minimal Clinically Important Difference (MCID) can offer a fresh perspective on assessing the extent to which a treatment provides real benefits for patients. The MCID for pain interference in patients with spinal conditions is a mean change of 8 or higher (Hung et al., 2018). A decline of 30% is considered the benchmark to identify meaningful improvement within the VAS arm and neck pain. (Bartels et al., 2017; Ostelo et al., 2008).

### 2.6 Successful outcome definition

Individual patient trajectories for clinical outcomes throughout time can vary widely. Therefore, we additionally dichotomized VAS arm pain and VAS neck pain for each patient individually into success versus non-success. For the VAS arm pain a good outcome is considered as equal or less than 2.5. Within the VAS neck pain, the outcome had to be equal or less than 3.5 (Mjåset et al., 2020).

### 2.7 Statistical analysis

Normally distributed variables are presented as mean ± standard deviation (SD). Differences between the three groups were analysed using linear mixed-effects models including a random intercept for each subject to account for repeated measures (Twisk, 2019). In a longitudinal data set, missing data is inevitable. There is no favorable approach in a mixed-model analysis with or without multiple imputations. However, the implementation of multiple imputation could make the results of mixed-model analyse unstable (Twisk et al., 2013). Therefore, multiple imputation was deemed unnecessary. The covariates age, gender, BMI, smoking behaviour and anxiety,/depression were chosen based on the experience of the senior authors.

Furthermore, each patient was classified as having a ‘successful’ or ‘unsuccessful’ outcome based on their VAS arm and VAS neck pain at baseline, 3, 6, 12 and 24 months after inclusion to evaluate individual differences. To assess potential differences in success percentages Pearsson Chi-Squared and Fisher's Exact tests were performed. A p-value of 0.05 or lower was considered statistically significant. IBM SPSS software, version 30.0, was used for all statistical analysis.

## 3 Results

### 3.1 Demographics, description of study population

A total of 1294 patients who underwent an ACDF procedure between January 2019 and November 2024 were identified and screened from the BWH. Of these, 460 were excluded due to the diagnosis cervical myelopathy and 300 patients were excluded because they underwent a multi-level procedure. Moreover, 2 patients were not eligible because they underwent surgery for malignancy and 5 patients because they underwent previous spine surgery. Finally, 45 patients were excluded due to insufficient clinical follow-up. Thus, a total of 482 patients with cervical radiculopathy that underwent an ACDF procedure from the BWH were included. Additionally, 88 patients from the CASINO trial (Netherlands cohort) were included to the standalone cage group. This leads to a sample size of 570 patients. Baseline characteristics, stratified on surgical device are demonstrated in Table 1. There were 414 patients who received a cage with plate (US), 54 patients who received cages with integrated screws (US) and 102 patients who received a standalone cage (86% Netherlands).

**Table 1:** Patient demographics; baseline characteristics of all patients in the cage with plate, cage with integrated screws and standalone cage group. The clinical outcome parameters are comparable among the three groups. SD stands for standard deviation. ∗ Is available for the US cohort only. For corresponding response rates per outcome, time point and treatment group, see Appendix A.

	Cage with Plate (n = 414)	Cage with Integrated Screws (n = 54)	Standalone Cage Merge (n = 102)	Standalone Cage Netherlands Cohort (n = 88)	Standalone Cage US Cohort (n = 14)
**Tabacco History (%)**					
**No**	259 (63)	29 (54)	58 (57)	50 (57)	8 (57)
**Yes, <1 pack/day**	107 (26)	14 (26)	10 (10)	7 (8)	3 (21)
**Yes, >1 pack/day**	48 (11)	11 (20)	34 (33)	31 (35)	3 (21)
**Pack Years∗, mean, SD**	4.8 ± 12	6.3 ± 11	3.9 ± 8		3.9 ± 8
**Male (%)**	187 (45)	24 (44)	43 (42)	36 (41)	7 (50)
**Mean Age, yrs (SD)**	57.5 ± 14	59.1 ± 12	49.8 ± 10	49.0 ± 10	53.7 ± 12
**Body Mass Index kg/m^2^ (SD)**	29.2 ± 6	28.9 ± 6	26.2 ± 4	25.9 ± 4	27.9 ± 4
**Anxiety-Depression∗**	51.3 ± 9	52.4 ± 10	54.8 ± 10		54.8 ± 10
**Arm Pain**	6.03 ± 2.6	5.58 ± 2.3	6.1 ± 2.4	6.1 ± 2.4	6.2 ± 2.0
**Neck Pain**	5.43 ± 2.8	5.05 ± 2.7	5.58 ± 2.7	5.59 ± 2.6	5.56 ± 2.7
**Audit-C∗**	2.8 ± 5	5.7 ± 9	3.3 ± 1		3.33 ± 1

### 3.2 Clinical outcomes

#### 3.2.1 Arm pain

The VAS arm pain in the standalone group showed comparable baseline mean values of 6.1 ± 2.4 in the Netherlands cohort standalone cage group and 6.2 ± 2.0 in the US standalone group (p = 0.868). At baseline, the mean values for arm pain were comparable in all groups: 6.0 ± 2.6 in the cage with plate group, 5.6 ± 2.3 in the cage with integrated screws group, and 6.1 ± 2.4 in the (merged) standalone cage group (p = 0.379). After two years of follow-up, these values decreased to 2.4 ± 2.4 (cage with plate), 2.3 ± 2.2 (cage with integrated screws) and 1.8 ± 2.7 (standalone group) (p = 0.491) (Table 2).

**Table 2:** Clinical outcome at baseline, 3, 6, 12 and 24 months of follow-up with standard deviations p-values for the between surgical treatment comparisons are given for each time points comparing the cage with plate, cage with integrated screws and standalone cage merge group, using a one-way ANOVA. For corresponding response rates per outcome, time point and treatment group, see Appendix A.

Time (months)		Cage with Plate (n = 414)	Cage with Integrated Screws (n = 54)	StandAlone Cage Merge (n = 102)	Stand Alone Cage Netherlands Cohort (n = 88)	Stand Alone Cage US Cohort (n = 14)	*p*
**Arm pain**	**0**	6.0 ± 2.6	5.6 ± 2.3	6.1 ± 2.4	6.1 ± 2.4	6.2 ± 2.0	0.379
	**3**	3.2 ± 2.9	3.1 ± 2.8	2.8 ± 3.1	2.7 ± 2.9	3.0 ± 3.1	0.771
	**6**	2.7 ± 2.5	2.0 ± 2.4	2.2 ± 2.6	2.2 ± 2.6	2.0 ± 2.6	0.122
	**12**	2.6 ± 2.6	3.1 ± 2.5	1.6 ± 2.3	1.7 ± 2.3	1.5 ± 2.3	0.013
	**24**	2.4 ± 2.4	2.3 ± 2.2	1.8 ± 2.7	1.9 ± 2.7	1.6 ± 2.6	0.491

**Neck pain**	**0**	5.4 ± 2.8	5.1 ± 2.7	5.6 ± 2.7	5.6 ± 2.6	5.6 ± 3.7	0.460
	**3**	3.9 ± 2.9	4.6 ± 2.7	3.6 ± 3.0	3.5 ± 2.9	4.7 ± 3.8	0.536
	**6**	3.1 ± 2.7	3.9 ± 3.0	2.8 ± 2.6	2.7 ± 2.4	3.4 ± 3.5	0.439
	**12**	3.2 ± 2.6	3.9 ± 2.4	2.1 ± 2.4	2.2 ± 2.4	1.3 ± 1.6	0.009
	**24**	3.5 ± 2.9	3.7 ± 2.6	1.8 ± 2.0	1.8 ± 2.0	1.5 ± 1.9	0.005

The differences between mean arm pain values at baseline and at 1 year follow-up exceeded the MCID of 30% in all groups: within the cage with plate group there was a 56.7% decline for the mean value of arm pain, which was 44.6% in the cage with integrated screws group and 70.5% in the standalone cage group. After 2 years of follow-up, the mean values for arm pain declined with 60.0% on average in the cage with plate group compared to baseline. The decline in the cage with integrated screws group was similar with 58.9%. In the standalone group, the largest decline of 70.5 % was noted (Table 2).

Likewise, linear mixed-effects models did not demonstrate significant differences regarding arm pain on the 10-point VAS scale. Overall mean differences were: 0.05 (p = 0.898) for cage with plate versus cage with integrated screws, 0.69 (p = 0.352) for cage with plate versus the standalone cage patients and 0.29 (p = 0.724) for the cage with integrated screws versus the standalone cage group (Table 3).

**Table 3:** Linear mixed-effects models including random intercepts, adjusted for baseline differences, age (p = 0.882), gender (p = 0.113), BMI (p = 0.171), smoking (p = 0.855), anxiety/depression (p = 0.031).

		Cage with Plate versus Cage with Integrated Screws			Standalone Cage (merge) versus Cage with Plate			Cage with Integrated Screws versus Standalone Cage		
		*B*	*95% CI*	*p*	*B*	*95% CI*	*p*	*B*	*95% CI*	*p*
**Pain Interference∗**	**Overall**	−1.13	−3.75; 1.49	0.397	0.86	−4.00; 5.72	0.730	0.42	−5.07; 5.91	0.877
	**3mo**	−2.31	−5.85; 1.23	0.200	−1.28	−7.67; 5.11	0.694	0.34	−6.09; 6.78	0.914
	**6mo**	−1.00	−4.43; 2.43	0.567	1.41	−4.64; 7.46	0.646	1.16	−5.16; 7.49	0.714
	**12mo**	−1.47	−5.14; 2.19	0.430	1.18	−5.70; 8.06	0.737	1.25	−5.65; 8.16	0.718
	**24mo**	−0.80	−4.75; 3.14	0.689	2.73	−5.62; 11.09	0.520	2.05	−5.85; 9.95	0.607

**Arm Pain**	**Overall**	0.05	−0.74; 0.84	0.898	0.69	−0.77; 2.16	0.352	0.29	−1.40; 1.99	0.724
	**3mo**	−0.11	−1.17; 0.96	0.845	−0.88	−2.86; 1.10	0.384	−1.13	−3.21; 0.95	0.281
	**6mo**	0.58	−0.47; 1.64	0.273	0.91	−0.86; 2.67	0.314	−0.05	−1.97; 1.87	0.957
	**12mo**	−0.59	−1.84; 0.67	0.360	2.02	−0.32; 4.36	0.090	2.15	−0.31; 4.63	0.085
	**24mo**	−0.08	−1.55; 1.38	0.913	2.29	−0.85; 5.44	0.152	2.38	−0.83; 5.90	0.144

**Neck Pain**	**Overall**	−0.59	−1.41; 0.23	0.157	0.17	−1.35; 1.69	0.829	0.16	−1.42; 1.74	0.836
	**3mo**	−0.68	−1.72; 0.36	0.199	−0.44	−2.41; 1.54	0.663	−0.18	−2.20; 1.85	0.861
	**6mo**	−0.64	−1.68; 0.39	0.222	−0.02	−1.82; 1.76	0.982	−0.04	−1.90; 1.82	0.964
	**12mo**	−0.58	−1.83; 0.66	0.358	0.59	−1.71; 2.88	0.616	0.47	−1.98; 2.92	0.704
	**24mo**	−0.11	−1.63; 1.41	0.886	1.90	−1.11; 4.91	0.216	2.04	−1.21; 5.28	0.214

Models are adjusted for baseline differences, age, gender, Body Mass Index, Smoking, Anxiety/Depression.

When dichotomizing the reported arm pain data in ‘successful’ and ‘unsuccessful’ for each individual, one year after inclusion, 53.0% (CI: 48.1; 57.7%) of the cage with plate group, 37.5% (CI: 24.1; 50.0%) of the cage with integrated screws group and 72.4% (CI: 63.7; 83.3%) of the standalone group had a successful outcome. Significant differences were observed between the cage with plate and standalone cage group (p = 0.014) and between the standalone cage group and cage with integrated screws (p = 0.010). Two years after inclusion, both the cage with plate and cage with integrated screws group demonstrated a modest increase of successful outcome, reaching 56.9 % (CI: 52.2; 61.6%) and 54.5 % (CI: 40.7; 68.5%) respectively. The standalone cage group reached a higher proportion of successful outcomes of 75.0% (CI: 66.6; 83.4%), the statistical significance between the groups however, was no longer present (Table 4).

**Table 4:** Percentage of patients with a successful outcome based on pre-established cut-off values for Patient-Reported Arm and Neck pain (0-10) success in three groups. Differences in groups were tested with a: a Fisher's exact test or b) a Pearsson's chi-squared test. ∗M stands for Months of follow-up. Additionally, the 95% Confidence Intervals are reported.

Time (months)	Success percentages*; 95% CI*			*p*	*p*	*p*
ARM Pain (≤2.5)	Cage with Plate*; 95% CI*	Cage with Integrated Screws*; 95% CI*	Standalone Cage (merge)*; 95% CI*	Cage with Plate vs Cage with Integrated Screws	Cage with Plate vs Standalone Cage	Standalone Cage vs Cage with Integrated Screws
0 M^+^	11.6% (8.7; 14.7%)	8.1% (1.9; 16.7%)	10.2% (4.9; 16.7%)	0.782^a^	0.706^a^	1.000^a^
**3 M**	52.7% (47.8; 57.5%)	43.5% (29.6; 57.4%)	57.1% (47.1; 66.7%)	0.407^b^	0.546^b^	0.261^b^
**6 M**	53.8% (49.0; 58.7%)	65.2% (51.9; 77.8%)	66.1% (56.9; 75.5%)	0.303^b^	0.096^b^	0.937^b^
**12 M**	53.0% (48.1; 57.7%)	37.5% (24.1; 50.0%)	72.4% (63.7; 83.3%)	0.244^b^	0.014^b^	0.010^b^
**24 M**	56.9% (52.2; 61.6%)	54.5% (40.7; 68.5%)	75.0% (66.6; 83.4%)	0.885^a^	0.056^b^	0.182^a^
**NECK pain (≤3.5)**						
**0 M^+^**	25.2% (21.0; 29.5%)	30.6% (18.5; 42.6%)	24.7% (16.7; 33.3%)	0.507^b^	0.893^b^	0.499^b^
**3 M**	49.7% (44.9; 54.6%)	45.8% (33.3; 59.3%)	52.4% (43.1; 61.8%)	0.723^b^	0.717^b^	0.585^b^
**6 M**	59.5% (54.8; 64.3%)	58.3% (44.4; 70.4%)	62.9% (52.9; 72.5%)	0.916^b^	0.642^b^	0.696^b^
**12 M**	61.2% (56.5; 65.9%)	26.7% (14.8; 38.9%)	79.3% (71.6; 87.3%)	0.014^a^	0.019^b^	<0.001^a^
**24 M**	58.9% (54.1; 63.5%)	44.4% (31.5; 57.4%)	85.7% (78.4; 92.2%)	0.483^a^	0.004^b^	0.015^a^

#### 3.2.2 Neck pain

At baseline, the mean values for neck pain were 5.4 ± 2.8 in the cage with plate group and 5.1 ± 2.7 in the cage with integrated screws group. A subdivision within the standalone group by cohort type revealed mean values of 5.6 ± 2.6 in the Netherlands cohorts' standalone cage group and 5.6 ± 3.7 (p = 1.000) in the US Standalone's group at baseline. After merging the two standalone cage cohorts, a mean value of 5.6 ± 2.7 was reached, which was comparable to the other two groups (p = 0.460). After two years of follow-up, mean neck pain declined to 3.5 ± 2.9 in the cage with plate group, 3.7 ± 2.6 in the cage with integrated screws group and 1.8 ± 2.0 (standalone group) (p = 0.005) (Table 2)

The cage with plate group reached the MCID of 30% both after 1 and 2 years of follow-up with a 40.7% and 35.2% lower mean value for neck pain. The cage with integrated screws group did not exceed the threshold with a change of 23.5% after 1 year and 27.5% after 2 years of follow-up compared to baseline. The standalone cage group mirrored the cage with plate group, demonstrating a 62.5% reduction in mean neck pain after 1 year, which further decreased with 67.9% after two years (Table 2).

In line with VAS arm pain, a linear mixed-models approach revealed no differences for neck pain between any of the groups in general over time. Overall mean differences were: −0.59 (p = 0.157) for cage with plate versus cage with integrated screws, 0.17 (p = 0.829) for cage with plate versus the standalone cage patients and 0.16 (p = 0.836) for the cage with integrated screws versus the standalone cage group (Table 3).

Similar to arm pain, the cut-off values for success revealed that one year after inclusion 61.2% (CI: 56.5; 65.9%) of the cage with plate group, 26.7% (CI: 14.8; 38.9%) of the cage with integrated screws group and 79.3% (CI: 71.6; 87.3%) of the standalone cage group had a successful outcome, while after two years these percentages changed to 58.9% (CI: 54.1; 63.5%), 44.4% (CI: 31.5; 57.5%) and 85.7% (CI: 78.4; 92.2%). After both one and two years, the standalone cage group showed significantly higher success percentages in comparison to the cage with plate group (p = 0.019; p = 0.004) and to the cage with integrated screws group (p < 0.001; p = 0.015).

#### 3.2.3 Pain interference (US cohort only)

Pain interference scores decreased within all groups, with the largest reduction observed in the first three months postoperatively. At baseline, the pain interference scores ranged from 64.3 ± 8 to 66.8 ± 6. After 24 months, these values decreased to 56.1 ± 9 in the cage with plate group, 57.3 ± 8 in the cage with integrated screws group and 53.9 ± 11 in the US Standalone cage group (p = 0.536) (Table 2).

In the cage with plate group, the MCID for pain interference (defined as a decrease of at least 8 points) was not attained at the one-year follow-up, with a mean decline of 7.0 points. At two years, however, the MCID was achieved, with a mean decline of 8.2 points. In contrast, patients treated with a cage with integrated screws did not reach the MCID at either follow-up, demonstrating mean declines of 4.5 points at one year and 6.7 points at two years. Notably, patients in the US standalone group achieved the MCID at both follow-up points, with mean declines of 8.1 points at one year and 12.9 points at two years (Table 2).

Linear mixed-effects models showed no significant differences in pain interference between the groups over time on a scale of 41.6 to 75.6. The estimated overall effects showed a mean difference of −1.13 (p = 0.397) between the cage with plate and cage with integrated screws group, 0.86 (p = 0.730) between the cage with plate and US standalone cage group and 0.42 (p = 0.877) in the comparison between cage with integrated screws and the US standalone cage group. The same trajectory was noted over a time period of 12 and 24 months (Table 3).

## 4 Discussion

The findings of our study indicate that additional cage fixation with plates or screws demonstrates no clear benefit in patient-reported outcomes in individuals with cervical radiculopathy undergoing single-level ACDF. Notably, in contrast to the other groups, the standalone cage group consistently achieved the MCID for pain interference, arm pain, and neck pain at both one- and two-year follow-up. Furthermore, the patients in this study experienced higher success rates for arm and neck pain when treated with a standalone cage in comparison to those receiving a cage with plate or a cage with integrated screws.

### 4.1 Generalizability of the study results

An interesting consideration is that most patients who received a standalone cage in our study were from Europe, whereas the groups treated with a cage and plate or a cage with integrated screws predominantly included patients from the United States. This raises the concern that differences in outcome might be influenced by regional surgical practices. The authors acknowledge that when patients are treated in different hospitals, differences will inevitably exist with respect to factors such as surgeon training and patients’ expectations. For this reason, the two standalone cage groups are compared with great caution. The Netherlands Standalone cage group was younger and had a lower average BMI compared to the US Standalone group, both of which occasionally have been associated with improved outcomes in ACDF surgery. Nonetheless, both variables were not significant within our analysis (p = 0.882 and p = 0.171). Moreover, our data showed that the two standalone cage subsets have comparable clinical outcomes (Table 2), regardless of whether they were from Europe or the United States. Although the subset from the US was small, comparing the subgroup with the Netherlands standalone group is particularly informative. As the clinical outcomes observed between these two subgroups are comparable, we cautiously conclude that regional differences are unlikely to explain the observed results.

Healthcare system–related factors, including implant availability, reimbursement policies, and the medico-legal environment, may also influence implant selection independently of clinical evidence. In particular, anterior plating may be favoured in certain regions due to perceived medico-legal risk mitigation or reimbursement incentives rather than demonstrated clinical superiority. These systemic factors further suggest that construct choice may reflect non-clinical drivers, underscoring the importance of evidence-based evaluation of fixation strategies.

### 4.2 Cage fixation and clinical outcomes

In the presence of comparable clinical outcomes across the three groups, a subsequent analytical step would be to assess and compare complication rates among the different treatment groups. A relevant differentiator between the constructs is dysphagia. An RCT by Zavras et al. (2022), comparing standalone cages to anterior plating systems in one- and two-level ACDF found that patients who received a cage with plate or a cage with integrated screws reported a significantly worse swallowing function at both 6 weeks and 6 months after surgery. This supports the hypothesis that both a cage with plate and a cage with integrated screws could possibly lead to damage to the overlying esophagus. Similar findings have been observed in multilevel ACDF by Wei Li et al. (2022), reporting significantly lower rates of dysphagia in the standalone group compared to the plating group. Although data on dysphagia was not collected in the current study due to its retrospective character, these findings are noteworthy, as reduced anterior soft tissue manipulation and a lower implant profile may play a role in the slightly better clinical outcomes we observed in the standalone group. It is important to acknowledge that in some regions, especially in the US, anterior plating may often be used as a precautionary measure rather than proven significant clinical benefit: despite the rarity of cage migration, fear of litigation plays an important role (Arif et al., 2024; Sharma and Sieg, 2022).

A recent study by Zhang et al. (2025), using zero-profile cages demonstrated that preservation of the anterior vertebral body edge significantly reduced cage subsidence, although final clinical outcomes remained comparable between groups. Subsequent methodological commentary on this work (De Simone et al., 2025) emphasized that cage subsidence should be interpreted as a multifactorial phenomenon, influenced not only by surgical technique but also by patient-related bone quality, implant material properties, and biomechanical interactions between the cage and fixation constructs. This broader biomechanical perspective highlights that construct stability and load distribution across the interbody device may play a key role in mitigating micromotion and implant-related complications following ACDF. These considerations underline that differences in fixation strategy may influence postoperative segmental stability and mechanical load sharing, which could partly explain variations in clinical symptom persistence observed between implant constructs.

The hypothesized minor long-term advantage of the standalone cage in comparison to the cage with plate and cage with integrated screws may be attributed to the mechanical behavior of the implants with screws. Although screw loosening generally is considered uncommon, the screws can theoretically be subjected to micromotion as long as intervertebral fusion is absent. This micromotion may lead to local irritation and increased mechanical stress on the vertebra, potentially contributing to recurrent or persistent radicular symptoms. Previous research has demonstrated that fusion between cervical vertebra after anterior discectomy takes at least several months (de Vries et al., 2025). During all this time micromotion may execute its irritating effect. Fusion is reported to be influenced by implant materials which also may have played a role in the observed outcomes. Titanium cages are associated with higher fusion rates, whereas PEEK cages, which are hydrophobic and less osteoconductive are less likely to promote fusion. Literature data however, reveal that these differences however are negligible (Goedmakers et al., 2022). Ideally, follow-up X-rays would have been available at the evaluated time points to assess fusion and position of the screws and implanted plated and cages to further substantiate these findings. However, due to the retrospective nature of this study, consistent and detailed data on these variables were unavailable, limiting the ability to explore their impact.

### 4.3 Strengths and limitations

Our study has multiple strengths. The inclusion of patients from two distinct healthcare systems in the United States and the Netherlands offers a unique and representative sample of ACDF procedures, which strengthens the generalizability of our findings. Moreover, having multiple follow-up timepoints allows us to get a better understanding of patient recovery throughout time. However, this study also includes limitations such as the lack of anxiety-depression scores in the Netherlands cohort. The data retrieved from the US treated patients revealed that this is a significant covariate (p = 0.031), which is in line with the current literature (Goedmakers et al., 2022), describing a strong association between mental health status and disability in single-level ACDF procedures. While there is a possibility that this introduced residual confounding, the consistency of the observed patterns across different outcomes and follow-up periods suggests that the overall conclusions of our study are unlikely to be significantly affected. Another limitation is the substantial differences in group sizes. In efforts to address this, 95% confidence intervals were calculated for all success percentages to provide a more accurate representation of the precision of our estimates across groups. Moreover, we performed additional sensitivity analyses using the Fisher's exact test to confirm our chi-squared results.

As a result of the retrospective nature of this study, data on fusion were not available, nor did we have access to detailed procedural information or data on common complications, which would ideally complement the clinical outcomes. This limitation is partly due to the study's retrospective design and partly attributable to clinical practice, in which patients often undergo surgery at one hospital but frequently receive follow-up care at external institutions, making it difficult to consistently obtain postoperative imaging. Nonetheless, the study provides an interesting and important perspective, addressing a relevant question in the treatment of ACDF. Future prospective research should aim to incorporate additional factors, such as dysphagia and fusion, to evaluate their potential impact on the (clinical) outcomes in the different groups.

Given that the comparison of mean clinical values through linear mixed-effects models did not show differences, but the individual-level success threshold revealed notable distinctions, underscores the complexity of evaluating treatment efficacy and highlights the need for a more personalized approach. This complexity arises from heterogeneity in patient factors such as baseline symptom severity and biological variability in healing processes. A plausible statistical explanation for the discrepancy is that within the linear mixed-effects models, averages across the entire cohort are assessed, which may conceal clinically meaningful improvements experienced by subgroups of patients. In contrast, dichotomized outcomes such as success rates, focus on individual patients’ outcomes which could be more sensitive to detecting treatment effects in specific patients. Furthermore, dichotomization helps mitigate the influence of outliers that might dilute group level comparisons. A logical follow-up step would be the development of a prediction model to identify which patients are most likely to benefit from specific treatment strategies. This observation aligns with the concept of responder analyses and patient-centered outcome evaluation frameworks, which emphasize clinically meaningful improvement at the individual level rather than average group-level changes. In heterogeneous populations, such as patients undergoing ACDF, mean-based analyses may obscure meaningful treatment effects experiences by subsets of patients, particularly when baseline severity and recovery trajectories vary widely. Therefore, success-rate analyses can provide complementary and clinically relevant information for shared decision-making, as they reflect the proportion of patients achieving outcomes that are meaningful in daily clinical practice.

## 5 Conclusion

In conclusion, our findings suggest that the choice of implant does not appear to substantially affect clinical outcomes over time at the group mean level. The standalone cage group demonstrated higher individual-level success-rates Given the frequency with which ACDF is and will be performed, it is essential to pay careful attention to these nuances in both clinical practice and future research, ideally through prospective studies that control for potential confounders.

## Ethical review committee statement

Approval for the project entitled was obtained from the Institutional Review Board (IRB) of the Brigham and Women's Hospital, Boston, MA, USA(IRB no 2015P002352). The requirement for informed consent was waived due to the retrospective design of the study, which involved no direct contact with human or animal subjects.

## Funding

This research did not receive any specific grant from funding agencies in the public, commercial, or not-for-profit sectors.

## Declaration of competing interest

The authors declare that they have no known competing financial interests or personal relationships that could have appeared to influence the work reported in this paper.

## Data Availability

The data presented in this study are available on request from the corresponding author; hereafter, an application must be submitted to the Medical Ethical Committee.

## References

[bib1] Arif H., Razzouk J., Bohen D., Ramos O., Danisa O., Cheng P., Cheng W. (2024). Analysis of reasons for medical malpractice litigation due to anterior cervical discectomy and fusion. World Neurosurg..

[bib2] Ban D., Liu Y., Cao T., Feng S. (2016). Safety of outpatient anterior cervical discectomy and fusion: a systematic review and meta-analysis. Eur. J. Med. Res..

[bib3] Bartels R.H.M.A., Donk R.D., Verhagen W.I.M., Hosman A.J.F., Verbeek A.L.M. (2017). Reporting the results of meta-analyses: a plea for incorporating clinical relevance referring to an example. Spine J. : official journal of the North American Spine Society.

[bib4] Buwaider A., El-Hajj V.G., MacDowall A., Gerdhem P., Staartjes V.E., Edström E., Elmi-Terander A. (2025). Machine learning models for predicting dysphonia following anterior cervical discectomy and fusion: a Swedish Registry Study. Spine J. : official journal of the North American Spine Society.

[bib5] Carlsson A.M. (1983). Assessment of chronic pain. I. Aspects of the reliability and validity of the visual analogue scale. Pain.

[bib6] Chang C.J., Liu Y.F., Hsiao Y.M., Huang Y.H., Liu K.C., Lin R.M. (2022). Comparison of anterior cervical discectomy and fusion versus artificial disc replacement for cervical spondylotic myelopathy: a metaanalysis. J. Neurosurg. Spine.

[bib7] De la Garza-Ramos R., Xu R., Ramhmdani S., Kosztowski T., Bydon M., Sciubba D.M., Wolinsky J.P., Witham T.F., Gokaslan Z.L., Bydon A. (2016). Long-term clinical outcomes following 3- and 4-level anterior cervical discectomy and fusion. J. Neurosurg. Spine.

[bib8] De Simone M., Amoroso E., Iaconetta G. (2025). Letter to the editor regarding "Does Bone Preservation at the Anterior Edge of the Vertebral Body Affect the Subsidence of Zero-Profile Cages After Anterior Cervical Discectomy and Fusion?". World Neurosurg..

[bib9] de Vries F.E., Gül A., Mesina-Estarrón I., Mekary R.A., Vleggeert-Lankamp C.L.A. (2025). Evaluation of bony fusion after anterior cervical discectomy: a systematic literature review and meta-analysis. Neurosurg. Rev..

[bib10] Elias E., Daoud A., Smith J., Elias C., Nasser Z. (2024). Assessing surgical outcomes for cage plate System versus stand-alone cage in anterior cervical discectomy and fusion: a systematic review and meta-analysis. World Neurosurg..

[bib11] Goedmakers C.M.W., van Beelen I., Komen F., van Zwet E.W., Peul W.C., Arts M.P., Vleggeert-Lankamp C.L.A. (2022). The impact of mental health on outcome after anterior cervical discectomy: cohort study assessing the influence of mental health using predictive modelling. Acta neurochirurgica.

[bib13] HealthMeasures (2025). https://www.healthmeasures.net/images/PROMIS/manuals/PROMIS_Anxiety_Scoring_Manual.pdf.

[bib14] HealthMeasures (2025). https://www.healthmeasures.net/images/PROMIS/manuals/PROMIS_Depression_Scoring_Manual.pdf.

[bib15] Hung M., Saltzman C.L., Kendall R., Bounsanga J., Voss M.W., Lawrence B., Spiker R., Brodke D. (2018). What are the MCIDs for PROMIS, NDI, and ODI instruments among patients with spinal conditions?. Clin. Orthop. Relat. Res..

[bib16] Huskisson E.C. (1974). Measurement of pain. Lancet.

[bib17] Kotkansalo A., Malmivaara A., Korajoki M., Korhonen K., Leinonen V. (2019). Surgical techniques for degenerative cervical spine in Finland from 1999 to 2015. Acta neurochirurgica.

[bib18] Li W., Zhan B., Jiang X., Zhou G., Li J., Wang Y. (2022). A randomized controlled study of two different fixations in anterior cervical discectomy of multilevel cervical spondylotic myelopathy. J. Orthop. Surg..

[bib19] Mjåset C., Zwart J.A., Goedmakers C.M.W., Smith T.R., Solberg T.K., Grotle M. (2020). Criteria for success after surgery for cervical radiculopathy-estimates for a substantial amount of improvement in core outcome measures. Spine J. : official journal of the North American Spine Society.

[bib20] Neifert S.N., Martini M.L., Yuk F., McNeill I.T., Caridi J.M., Steinberger J., Oermann E.K. (2020). Predicting Trends in Cervical Spinal Surgery in the United States from 2020 to 2040. World Neurosurg..

[bib21] Nemoto O., Kitada A., Naitou S., Tachibana A., Ito Y., Fujikawa A. (2015). Stand-alone anchored cage versus cage with plating for single-level anterior cervical discectomy and fusion: a prospective, randomized, controlled study with a 2-year follow-up. European journal of orthopaedic surgery & traumatology : Orthop. Traumatol..

[bib22] Oliver J.D., Goncalves S., Kerezoudis P., Alvi M.A., Freedman B.A., Nassr A., Bydon M. (2018). Comparison of outcomes for anterior cervical discectomy and fusion with and without anterior plate fixation: a systematic review and meta-analysis. Spine.

[bib23] Ostelo R.W., Deyo R.A., Stratford P., Waddell G., Croft P., Von Korff M., Bouter L.M., de Vet H.C. (2008). Interpreting change scores for pain and functional status in low back pain: towards international consensus regarding minimal important change. Spine.

[bib24] Pereira E.A., Chari A., Hempenstall J., Leach J.C., Chandran H., Cadoux-Hudson T.A. (2013). Anterior cervical discectomy plus intervertebral polyetheretherketone cage fusion over three and four levels without plating is safe and effective long-term. J. Clin. Neurosci. : official journal of the Neurosurgical Society of Australasia.

[bib25] Rao R.D., Gourab K., David K.S. (2006). Operative treatment of cervical spondylotic myelopathy. J Bone Joint Surg Am.

[bib26] Safdar A., Headley B., Rommelman M., Haseeb A., Motiei-Langroudi R. (2023). The effect of interbody cage parameters on the rate of subsidence in single-level anterior cervical discectomy and fusion (ACDF): a retrospective analysis of 98 patients. Cureus.

[bib27] Schuller W., Terwee C.B., Terluin B., Rohrich D.C., Ostelo R.W.J.G., de Vet H.C.W. (2023). Responsiveness and minimal important change of the PROMIS pain interference Item bank in patients presented in musculoskeletal practice. J. Pain.

[bib28] Sharma M., Sieg E. (2022). Mediastinal migration of standalone cage-plate construct following multilevel anterior cervical diskectomy and fusion. World Neurosurg..

[bib29] Simões de Souza N.F., Broekema A.E.H., Reneman M.F., Koopmans J., van Santbrink H., Arts M.P., Burhani B., Bartels R.H.M.A., van der Gaag N.A., Verhagen M.H.P., Tamási K., van Dijk J.M.C., Groen R.J.M., Soer R., Kuijlen J.M.A., on behalf of the FACET investigators (2024). Posterior cervical foraminotomy compared with anterior cervical discectomy with fusion for cervical radiculopathy: Two-Year results of the FACET randomized noninferiority Study. J. Bone Jt. Surg. Am. Vol..

[bib30] Simon C.B., McCabe C.J., Matson T.E., Oliver M., Bradley K.A., Hallgren K.A. (2024). High test-retest reliability of the Alcohol Use Disorders Identification Test-Consumption (AUDIT-C) questionnaire completed by primary care patients in routine care. Alcohol, clinical & experimental research.

[bib31] Sommaruga S., Camara-Quintana J., Patel K., Nouri A., Tessitore E., Molliqaj G., Panchagnula S., Robinson M., Virojanapa J., Sun X., Melnikov F., Kolb L., Schaller K., Abbed K., Cheng J. (2021). Clinical outcomes between stand-alone zero-profile spacers and cervical plate with cage fixation for anterior cervical discectomy and fusion: a retrospective analysis of 166 patients. J. Clin. Med..

[bib32] Takhelmayum Umesh, Acharya s, Chahal r.s, Kalra K.L., Gupta Pravin, Palukuri Nagendra (2019). Integrated screws with cage spacer system in the treatment of cervical spine degenerative disease with a minimum follow-up of 2 years. Journal of Orthopedics, Traumatology and Rehabilitation.

[bib33] Twisk J.W.R. (2019).

[bib34] Twisk J., de Boer M., de Vente W., Heymans M. (2013). Multiple imputation of missing values was not necessary before performing a longitudinal mixed-model analysis. J. Clin. Epidemiol..

[bib35] Vaishnav A.S., Saville P., McAnany S., Patel D., Haws B., Khechen B., Singh K., Gang C.H., Qureshi S.A. (2019). Predictive factors of postoperative dysphagia in single-level anterior cervical discectomy and fusion. Spine.

[bib36] Vleggeert-Lankamp C.L.A., Janssen T.M.H., van Zwet E., Goedmakers C.M.W., Bosscher L., Peul W., Arts M.P. (2019). The NECK trial: effectiveness of anterior cervical discectomy with or without interbody fusion and arthroplasty in the treatment of cervical disc herniation; a double-blinded randomized controlled trial. Spine J. : official journal of the North American Spine Society.

[bib37] Zavras A.G., Nolte M.T., Sayari A.J., Singh K., Colman M.W. (2022). Stand-Alone cage versus anterior plating for 1-Level and 2-Level anterior cervical discectomy and fusion: a randomized controlled trial. Clinical spine surgery.

[bib38] Zhang B., Kong Q., Feng P., Liu J., Ma J. (2025). Does bone preservation at the anterior edge of the vertebral body affect the subsidence of zero-profile cages after anterior cervical discectomy and fusion?. World Neurosurg..

