# Supplementary material for: Is outcome of anterior cervical discectomy for cervical radiculopathy influenced by securing the intervertebral cage?
Source: Brain Spine. 2026 Apr 12;6:106039. doi: 10.1016/j.bas.2026.106039 (PMC13094434; doi:10.1016/j.bas.2026.106039)
Supplement: Multimedia component 1 [file mmc1.docx]

**Appendix A: Response Rate (%) per clinical outcome, group and time point**

| **Time (months)** | **Outcome** | **Cage with Plate (n=414)** | **Cage with Integrated Screws (n=54)** | **Standalone Cage NL Cohort (n=88)** | **Standalone Cage US Cohort (n=14)** |
| --- | --- | --- | --- | --- | --- |
| 0 | Pain Interference | 100% | 100% |  | 100% |
| 3 | Pain Interference | 78% | 72% |  | 71% |
| 6 | Pain Interference | 76% | 71% |  | 71% |
| 12 | Pain Interference | 79% | 78% |  | 79% |
| 24 | Pain Interference | 82% | 74% |  | 71% |
|  |  |  |  |  |  |
| 0 | Arm Pain | 100% | 100% | 100% | 100% |
| 3 | Arm Pain | 73% | 69% | 95% | 71% |
| 6 | Arm Pain | 75% | 71% | 94% | 71% |
| 12 | Arm Pain | 86% | 81% | 92% | 79% |
| 24 | Arm Pain | 72% | 74% | 90% | 71% |
|  |  |  |  |  |  |
| 0 | Neck Pain | 100% | 98% | 100% | 100% |
| 3 | Neck Pain | 71% | 68% | 94% | 79% |
| 6 | Neck Pain | 68% | 71% | 93% | 79% |
| 12 | Neck Pain | 79% | 80% | 91% | 71% |
| 24 | Neck Pain | 81% | 73% | 89% | 71% |
