# Supplementary material for: Is outcome of anterior cervical discectomy for cervical radiculopathy influenced by securing the intervertebral cage?
Source: Brain Spine. 2026 Apr 12;6:106039. doi: 10.1016/j.bas.2026.106039 (PMC13094434; doi:10.1016/j.bas.2026.106039)
Supplement: Multimedia component 2 [file mmc2.pdf]

Approval for the project entitled *'Is outcome of Anterior Cervical Discectomy for Cervical Radiculopathy influenced by securing the Intervertebral Cage?'* was obtained from the institutional review board.

PARTNERS HEALTHCARE
FOUNDED BY BRIGHAM AND WOMEN'S HOSPITAL
AND MASSACHUSETTS GENERAL HOSPITAL

Partners Human Research Committee
116 Huntington Avenue, Suite 1002
Boston, MA 02116
Tel: (617) 424-4100
Fax: (617) 424-4199

Initial Review: Notification of IRB Approval/Activation
Protocol #: 2015P002352/BWH

Date: November 5, 2015

To: Timothy Smith
BWH
Neurosurgery

From: Partners Human Research Committee
116 Huntington Avenue, Suite 1002
Boston, MA 02116

Title of Protocol:	Diagnosis and management of rare CNS lesions: identifying differentiating features and predictors of improved outcomes, in order to improve quality of care.
Sponsor/Funding Support:	None
IRB Review Type:	Expedited
Expedited Category/ies:	(5)
IRB Approval Date:	11/5/2015
Approval Activation Date:	11/5/2015
IRB Expiration Date:	11/5/2017

This project has been reviewed by BWH IRB . During the review of this project, the IRB specifically considered (i) the risks and anticipated benefits, if any, to subjects; (ii) the selection of subjects; (iii) the procedures for obtaining and documenting informed consent; (iv) the safety of subjects; and (v) the privacy of subjects and confidentiality of the data.

Please note that if an IRB member had a conflict of interest with regard to the review of this project, consistent with IRB policies and procedures, the member was required to leave the room during the discussion and vote on this project except to provide information requested by the IRB.

The IRB has reviewed and approved the following: Health/ Medical Records.

As Principal Investigator, you are responsible for ensuring that this project is conducted in compliance with all applicable federal, state and local laws and regulations, institutional policies, and requirements of the IRB, which include, but are not limited to, the following:

1. Submission of any and all proposed changes to this project (e.g., protocol, recruitment materials, consent form, status of the study, etc.) to the IRB for review and approval prior to initiation of the change(s), except where necessary to eliminate apparent immediate hazards to the subject(s). Changes made to eliminate apparent immediate hazards to subjects must be reported to the IRB as an unanticipated

Partners Human Research Committee
116 Huntington Avenue, Suite 1002
Boston, MA 02116
Tel: (617) 424-4100
Fax: (617) 424-4199

problem.

2. Submission of continuing review submissions for re-approval of the project prior to expiration of IRB approval and a final continuing review submission when the project has been completed.
3. Submission of any and all unanticipated problems, including adverse event(s) in accordance with the IRB's policy on reporting unanticipated problems including adverse events.
4. Obtaining informed consent from subjects or their legally authorized representative prior to initiation of research procedures when and as required by the IRB and, when applicable, documenting informed consent using the current IRB approved consent form(s)with the IRB-approval stamp in the document footer.
5. Informing all investigators and study staff listed on the project of changes and unanticipated problems, including adverse events, involving risks to subjects or others.
6. When investigator financial disclosure forms are required, updating your financial interests in Insight and for informing all site responsible investigators, co-investigators and any other members of the study staff identified by you as being responsible for the design, conduct, or reporting of this research study of their obligation to update their financial interest disclosures in Insight if (a) they have acquired new financial interests related to the study and/or (b) any of their previously reported financial interests related to the study have changed.

The IRB has the authority to terminate projects that are not in compliance with these requirements.

Questions related to this project may be directed to Fred Syllien, FSYLLIEN@PARTNERS.ORG, 617-424-4124.

CC: Julian Bryan Iorgulescu, BWH - Pathology, Co-Investigator

Sagerian, Andrew

From: Sagerian, Andrew
Sent: Monday, September 11, 2023 2:44 PM
To: Smith, Timothy R.,MD, , MPH
Cc: Gerstl, Jakob; Shea, Heather E.; Carter, Heather; Research Information Security
Subject: RISO Approval Letter 2015P002352 AME168

Dr. Smith:

This email is to inform you that the data security review of your IRB protocol 2015P002352 AME168 has been completed and approved. Approval is predicated upon the fact that the following points assessed below are true and remain true during the study lifecycle.

- All study activities involving technology and data sharing with external collaborators prior to **AME168** have been approved by MGB IRB without RISO review and are outside of review scope
- Amendment scope:
  - Acceptance of de-identified retrospective clinical data from the following outside institutions:
    - MD Anderson Cancer Center
    - University of Alabama, Birmingham
    - Karolinska Institute (Sweden)
    - Uppsala University (Sweden)
- PI will re-approach RISO if it is determined that a data protection regulation reaches BWH extraterritorially
  - For any international data, study team should use systems that are approved by MGB for international data
    - PI will continue to work with RISO/BH RDP, as appropriate
  - BWH study will not be contacting or providing goods/services to data subjects in connection with this study
  - No results of this study will be returned to data subjects
- In-scope data is limited to de-identified clinical data on the lesions being studied
- BWH study team will receive and store data using [MGB Dropbox for Business]
  - Study team will use systems approved by MGB as compliant to store data from international sites
    - MGB Dropbox Business is approved for storage of international data
    - Study team will contact BH RDP/RISO with any questions
  - In-scope sites will give BWH study team access only to data requested in connection with this study
  - Data received will be from retrospective collection of medical records or gathered from clinical care
    - Because of the retrospective collection, no consent form will be used
      - BWH study staff will not have contact with patients from the external sites in connection with this study
      - Data was obtained from medical records, i.e., retrospective data acquired within clinical routine
- Study staff will ensure any required agreements, including research agreements, are fully executed, as applicable, before any data/sample sharing begins
  - Create a Data Use Agreement Record in Insight for MGB Contracting Offices to review and make an appropriate determination on agreement type
  - Appropriate MGB Contracting Office will ensure the necessary language pertaining to the transfer of data from in-scope sites to BWH are incorporated within
    - Appropriate MGB Contracting Office should assess applicability of any data protection regulation in connection with the use case, as appropriate and required

        - MGB Contracting Office will confer with OGC on applicability and agreement terms as needed
    - Study will re-approach RISO/BH RDP if it is determined a data protection regulation reaches BWH extraterritorially
    - Suggested Infosec Contractual Language Guidance should be followed (Infosec Contractual Language Guidance)
        - Study staff should confer with their assigned agreement associate to ensure baseline information security language is included in the agreement, as appropriate
    - Any data sharing should utilize Mass General Brigham approved methods (i.e., Mass General Brigham Dropbox Business, Mass General Brigham OneDrive for Business, or Mass General Brigham Secure File Transfer):
        - Email will not be used
    - No further sharing of the data will take place once received by BWH
        - Results will not be returned to data subjects by BWH nor by the originating sites
- Any emailing will comply with IRB and hospital policies and procedures, including MGB's Standards for Securing Electronic Communications:
    - Study staff will use their MGB email credentials, no personal or non-MGB email addresses (i.e., Gmail, etc.)
    - Send Secure will be used to encrypt email messages with Confidential data sent to email addresses outside of Mass General Brigham
    - Confidential data will not be included in email subject line
    - The blind copy function (BCC) must be used when sending to more than one patient/research subject, to protect the confidentiality of recipient
    - Department name or service in email signature or other information from which a health diagnosis or condition could be inferred will not be included in email body
    - IRB and hospital policies and procedures for emailing with subjects/prospective subjects will be followed (i.e. Procedure: Requests to Receive Unencrypted Email)
- Minimum necessary principle will always be followed
- Any de-identification of data will be conducted in compliance with hospital's De-Identification Policy and relevant study staff trained on policy:
    - Subject will have a unique identifier
    - Only approved and credentialed study staff will have access
    - Any linking file (i.e., subject key code) is kept secure always
    - BWH study staff will not have access to subject key codes or linking files from any of the other in-scope sites
- Any paper study records will be stored in locked cabinets/office at study site
- For any web portal access:
    - Data will only be accessed through the web portal
    - Data will always be encrypted in transit
    - HTTPs is enforced over HTTP
    - TLS version in use is 1.2 or superior
    - Weak ciphers are disabled
    - Modern ciphers are configured as preferred
    - Server ciphers preferred over client's
    - Certificates are valid and issued by a publicly-trusted certificate authority
    - Modern Internet browsers must be used to access
    - The web portal will be accessed only from MGB standard devices
    - Access to web portals by study staff require a username and password.
        - Passwords must be a minimum of 8 characters
        - Passwords must be alphanumeric, containing at least one of each.
        - Cannot reuse 5 previous passwords.

        - Passwords must be changed immediately if either the password or the system is or may be compromised.
        - Passwords must not be displayed in clear text when they are being input into an application.
        - Passwords must be changed every 180 days
        - Users must be uniquely identified; no shared or group accounts without security authorization
        - Passwords may not be shared.
    - Passwords should not be the same used for logging into MGB workstations/laptops unless single sign-on is implemented.
    - Access should be reviewed (and revoked) as study staff changes or when access is no longer needed
- Access to any research data, web portals, applications, etc. must be done from workstations and/or laptops configured to be compliant with MGB workstation policies;
    - Password requirements:
        - Required minimum length of 8 characters.
        - Passwords must be alphanumeric, containing at least one of each.
        - Cannot reuse 5 previous passwords.
        - Passwords must be changed immediately if either the password or the system is or may be compromised
        - Passwords must not be displayed in clear text when they are being input into an application.
        - Passwords must be changed every 180 days
        - Users must be uniquely identified, no shared or group accounts without security authorization
        - Passwords may not be shared
        - User access must be terminated immediately upon termination or change of responsibilities
    - Encryption at rest is in place
    - Up-to-date malware protection including antivirus, spyware detection and removal tools
    - Local firewall is enabled
    - Manufacturer supported operating system with, with up-to-date patches
    - Crowdstrike End Point protection installed or other advanced end point security solution that is acceptable to MGB (i.e., Symantec Endpoint Protection)
    - Network Access Control agent installed (ForeScout)
    - Any Macs must be enrolled in PEAS

Please let me know if you have any questions. This email can be forwarded or printed for your response to the IRB. Thank you.

Best,
Andrew

Andrew Sagerian
Research Information Security Analyst I
Brigham and Women's Hospital | Research Data Protection Office
399 Revolution Drive, Somerville, MA 02145
