# Supplementary material for: Is outcome of anterior cervical discectomy for cervical radiculopathy influenced by securing the intervertebral cage?
Source: Brain Spine. 2026 Apr 12;6:106039. doi: 10.1016/j.bas.2026.106039 (PMC13094434; doi:10.1016/j.bas.2026.106039)
Supplement: Multimedia component 3 [file mmc3.pdf]

Medisch-Ethische Toetsingscommissie
Leiden | Den Haag | Delft

commissie METC-LDD
postzone P5-P
Mw.mr. M. van Loon
telefoon (071) 526 3241 Of (071)5266963
e-mail metc-ldd@lumc.nl
onze referentie **P12.031**/ML/ib
uw referentie
Ccmo ref NL39403.058.12
datum 27 juni 2019
onderwerp **Besluit beoordeling amendement NL39403.058.12**

aan De weledelzeergeleerde vrouwe
dr. C.L.A.M. Vleggeert - Lankamp
afdeling Neurochirurgie
postzone **J-11-R, alhier**

Geachte mevrouw,

Hierbij zend ik u het besluit van de METC Leiden Den Haag Delft (hierna: METC LDD) inzake het onderzoeksprotocol getiteld: **"CASINO: Cervical surgical noncervical; een gerandomiseerd doelmatigheidsonderzoek naar de chirurgische behandeling van de cervicale HNP" (NL39403.058.12)**.

De METC LDD verleent goedkeuring aan het amendement in genoemd onderzoek. Voor de overwegingen verwijs ik u naar het bijgevoegde besluit.

Wij verzoeken u alle bij de uitvoering van het onderzoek betrokken partijen van het besluit op de hoogte te brengen

Vertrouwend u hiermee voldoende te hebben geïnformeerd.

Met vriendelijke groet,
namens de METC Leiden Den Haag Delft,

Mw. mr. M. van Loon
Secretaris

cc: prof. dr. W.C. Peul, Neurochirurgie, LUMC, Leiden
drs. S. van Geest, Neurochirurgie, LUMC, Leiden
dr. F.R. Gärtner, Medische Besliskunde, LUMC, Leiden
mr. G.C.M. Lafeber, Neurochirurgie, LUMC, Leiden
CCMO d.m.v. upload in ToetsingOnline (NL39403.058.12)

Medisch-Ethische Toetsingscommissie
Leiden | Den Haag | Delft

BESLUIT
Beoordeling amendement

NL nummer	NL39403.058.12	METC-nummer	P12.031
Titel onderzoek	"CASINO: Cervical surgical noncervical; een gerandomiseerd doelmatigheidsonderzoek naar de chirurgische behandeling van de cervicale HNP"		

Contactgegevens: dr. C.L.A.M. Vleggeert - Lankamp, Neurochirurgie, LUMC, Leiden
Verrichter: LUMC, Leiden

Besluit
De medisch-ethische toetsingscommissie Leiden Den Haag Delft (hierna: METC LDD) heeft zich, op grond van artikel 2, tweede lid, sub a van de Wet medisch-wetenschappelijk onderzoek met mensen (WMO), beraden over het amendement behorend bij bovengenoemd onderzoeksdossier.

De METC LDD heeft eerder een positief oordeel gegeven over het onderzoeksdossier in de volgende centra:
- het LUMC te Leiden (hoofdonderzoeker: Dr. C.L.A.M. Vleggeert - Lankamp)
- UMC St. Radboud te Nijmegen (hoofdonderzoeker : R.H.M.A. Bartels);
- Martini Ziekenhuis te Groningen (hoofdonderzoeker: D.L.M. Oterdoom);
- Maasstad Ziekenhuis te Rotterdam (hoofdonderzoeker: B. Kuijper);
- Canisius-Wilhelmina Ziekenhuis te Nijmegen (hoofdonderzoeker: H.D. Boogaarts);
- St. Elisabeth Ziekenhuis te Tilburg (hoofdonderzoeker: E.C. Lamers);
- Reinier de Graaf Groep te Delft (hoofdonderzoeker: H.J. Gilhuis);
- Alrijne/Rijnland Ziekenhuis te Leiderdorp (hoofdonderzoeker: M.J.T. Verstegen);
- St. Antonius ziekenhuis te Nieuwegein (hoofdonderzoeker: P.H. Wessels);
- Medisch Centrum Haaglanden te Den Haag (hoofdonderzoeker: N. van Dijk);
- Haga ziekenhuis te Den Haag (hoofdonderzoeker: H. Koppen);
- Diakonessenhuis te Utrecht (hoofdonderzoeker: R.C.J.M. Donders);
- Vlietland ziekenhuis te Schiedam (hoofdonderzoeker: C.L. Alblas)

De commissie oordeelt positief over het amendement.

Documenten
Het besluit is gebaseerd op de documenten die in bijlage 1 zijn vermeld.

Achtergrond
Op 21-09-2018 is het amendement ter beoordeling bij de METC LDD ingediend. Het amendement heeft betrekking op het aanpassen van de studie-opzet (in verband met een tegenvallende inclusie) en op het toevoegen van een vragenlijst.

Het amendement is besproken in de vergaderingen van 27-11-2018 en 12-02-2019: zie bijlage 2 voor de samenstelling van de commissie ten tijde van de vergadering van 12-02-2019.

Overwegingen
De METC LDD is van oordeel dat aan alle voorwaarden in artikel 3 van de WMO is voldaan. De belangrijkste vragen die in het kader van de beoordeling gesteld werden hadden betrekking op de vraag of de aangepaste studie-opzet als amendement of als geheel nieuwe studie gezien moet worden. Daarnaast heeft de commissie gevraagd de proefpersoneninformatiebrief op gespecificeerde punten aan te passen. Nu de gestelde vragen naar tevredenheid zijn beantwoord

en de documentatie conform instructie is aangepast, gaat de commissie over tot het nemen van een positief besluit.

Naar de mening van de commissie heeft het amendement geen directe consequenties voor lokale uitvoerbaarheidsaspecten.

Ten slotte wijst de METC LDD u op de verplichtingen die bij het oorspronkelijke positieve besluit zijn vermeld.

Hoogachtend,
Namens de METC Leiden Den Haag Delft,

Mw.mr. M. van Loon,
secretaris

Leiden, 27 juni 2019

Beroepsprocedure
Tegen dit besluit kan een belanghebbende op grond van artikel 23 van de WMO binnen zes weken na de dag waarop het besluit is bekend gemaakt, administratief beroep instellen bij de Centrale Commissie Mensgebonden Onderzoek (CCMO). Het beroepschrift dient u te adresseren aan CCMO, Postbus 16302, 2500 BH Den Haag.

Bijlage 1

Documenten

- A1 Aanbiedingsbrief amendement d.d. 20-09-2018
- A1 Beoordelingsbrief CME d.d. 08-10-2018; Antwoord indiener d.d.19-11-2018; beoordelingsbrief CME d.d. 06-12-2018; antwoordbrief indiener d.d. 16-01-2019
- B1 ABR formulier versie 14 d.d. 19-11-2018
- C1 Onderzoeksprotocol d.d. 16-01-2019
- E1 Informatiebrief voor proefpersoon met toestemmingsverklaring CASINO cohort GL versie 1 d.d. 01-2019
- F1 vragenlijst WOMAC versie 1 d.d. 20-11-2018
- K1 Goedkeuring WetCie neurochirurgie d.d. 08-12-2018

Bijlage 2

Samenstelling METC Leiden Den Haag Delft

Voorzitters
- Prof dr. A. Dahan, voorzitter, anesthesioloog
- Prof. dr. M. de Vries, vicevoorzitter, kinderarts

Artsen
- Dr. U.A. Badrising, neuroloog
- Dr. M. Haak, gynaecoloog-perinatoloog
- Dr. E. Kapiteijn, internist-oncoloog
- Dr. G.J. Liefers, oncologisch chirurg
- Prof. dr. E. Lopriore, kinderarts-neonatoloog
- Prof. dr. A.B. te Pas, kinderarts-neonatoloog
- Dr. A.J. Peeters, reumatoloog
- Dr. M.E. Tushuizen, MDL-arts
- Prof. dr. M.J.H. Wermer, neuroloog

Methodologen
- Prof. dr. R.H.H. Groenwold
- Prof. dr. H. Putter
- Dr. E.W. van Zwet

Ethici
- Dr. M. Houtlosser
- Dr. D.P. Touwen
- Prof. dr. M. de Vries

Juristen
- Dr. mr. M. Eijkholt
- Mr. M.F. van der Mersch
- Mr. C.E. Philips- Santman

Klinisch farmacologen / ziekenhuisapothekers
- Prof. dr. H.J. Guchelaar
- Dr. J.J. Swen
- Dr. J. Zwaveling
